# Gleanings from French Journals

**Published:** 1889-10

**Authors:** A. Dagenais


### GLEANINGS FROM FRENCH JOURNALS.

By DR. A. DAGENAIS.

M. Tarnier presented a case, at the meeting of the Academy of Medicine, of a woman having an extrauterine pregnancy dating from 1856. This pregnancy had gone full term, the beating of the fetal heart could be heard, but the accoucheur, being certain of an extra-uterine pregnancy, asked the advice of Paul Dubois upon the necessity of a prompt intervention, who prefers not to perform laparatomy, and let things as they were. After a few days, the motion of the fetus stopped, the pain, which had been violent, disappears suddenly, and the patient got up soon after. In 1859, she consulted Nêlaton, who refused to operate. A short time ago, M. Tarnier made an examination, which demonstrated a sub umbilical tumor, constituted by the fetus. This tumor is divided in two parts, corresponding to the head and trone of the fetus.

---

Every year the French Red-Cross increases its material of ambulance. This year 100,000 francs have been given to that effect, which carries to date the value of this material to a million of francs.

---

A New Process of Expulsion of Foreign Bodies Swallowed.—A simple process which is actually in use at the clinic of Prof. Billroth, and which is known under the name of "*Cure de pommes de terre*," has already been indicated by Cameron (of Glasgow) in 1887. The patients are told to eat a great quantity of potatoes, which produces a uniform distention of the intestinal tube, and forces the expulsion of the foreign body by the natural channels.—*L'Abeille Medicale.*

